# Sensors Network and Wearables for People Activities and Wellbeing Monitoring

**DOI:** 10.3390/s26082475

**Published:** 2026-04-17

**Authors:** Sara Casaccia, Gloria Cosoli

**Affiliations:** 1 Department of Industrial Engineering and Mathematical Sciences, Università Politecnica Delle Marche, v. Brecce Bianche 12, 60131 Ancona, Italy; s.casaccia@staff.univpm.it; 2 Department of Theoretical and Applied Sciences, eCampus University, v. Isimbardi 10, 22060 Novedrate, Italy

## 1. Introduction

In recent decades, distributed sensor network and wearable sensors have known an unprecedented development [1,2,3]. This has allowed researchers to study human activities [4,5,6] and behavioral parameters [7,8], exploiting low-cost devices preserving privacy and guaranteeing non-intrusivity. The definition of data processing pipelines plays a pivotal role in this context, and Artificial Intelligence (AI)-based algorithms have made great strides, supporting the effective analysis of multimodal data [9], both for classification and prediction purposes [10,11,12,13]. The human-in-the-loop paradigm [14] can now be considered essential, given that decision-making processes can benefit from AI, but humans must be always the final decision makers since the responsibility of decisions and, consequently, actions is fundamental and cannot be transferred to AI algorithms, whatever the application field.

At present, monitoring people’s activities and behavior [15] is a widespread practice in several contexts, from personalized comfort assessment [16] to general health status monitoring [17] and wellbeing [15], and in diverse environments, both indoor and outdoor.

## 2. Overview of the Contributions

This Special Issue confirms what is stated above as well as the multidisciplinarity and wide applicability of distributed sensor networks and wearables for activity monitoring. The contributions present innovative solutions and advanced signal processing techniques as well as analyses and considerations on such systems’ performance and characteristics.

Building on the general relevance of wearable and distributed sensor systems, the contributions in this Special Issue can be organized into four key application domains. The first domain addresses personalized comfort assessment, exploring how physiological, environmental, and perceptual data can be integrated through wearable devices to better understand individual comfort [18,19]. The second domain focuses on sleep monitoring, where continuous, non-intrusive sensing allows the analysis of sleep patterns and quality in real-life conditions [20,21]. The third domain concerns Ambient Assisted Living (AAL) and frailty monitoring [22], highlighting how sensors can support mobility assessment, fall detection [23], and independent living for older adults or individuals with neurodegenerative and mobility-impairing conditions [24]. Finally, the fourth domain investigates human behavior, perception, and functional abilities, showing how wearable sensors capture physiological responses, social interactions [25,26], and motor performance in everyday scenarios. The key contributions in each of these areas are summarized below.

Comfort assessment (and especially personalized comfort assessment) is one of the most interesting application fields for such techniques. In this context, multi-sensor approaches are of interest [27,28], since both environmental and physiological data, together with perceptual information, can contribute to a comprehensive assessment of personalized comfort. The following contributions address different aspects related to environmental monitoring and physiological sensing in this context.

In Contribution 1, the authors investigate how contact pressure and activity level affect PPG signal quality, showing that even subtle physiological and interaction factors can impact on the sensor performance. Hence, not only data processing techniques but also hardware design have a relevant impact on the reliability of data used in personalized comfort assessment. Optimizing the design since its early phase can significantly enhance measurement accuracy (mean absolute percentage errors—MAPEs—<7% are also obtained at a high activity intensity level), thus providing good quality data to Personal Comfort Models (PCMs).

Contribution 2 presents a wearable sensor node for measuring air quality through a citizen science approach, highlighting that integrating fine-grained environmental measurements with user behavior can provide deeper insights into individual comfort conditions. High correlation (R^2^ = 0.75) and limited MAPE (0.54 µg/m^3^) are reported, together with a high satisfaction level among participants (>70%, considering seniors, commuters, and students). Such a sensing node can provide reliable information, thus empowering citizens and supporting the definition of robust environmental policies.

In Contribution 3, the authors examine CO_2_ concentration, temperature, and humidity in Italian university classrooms over different seasons, demonstrating that a comprehensive analysis of their interaction can provide relevant insights on the occupants’ comfort. Indoor Air Quality (AIQ) and thermal comfort are related to these environmental quantities, which can significantly impact on overall wellbeing and performance.

Another relevant application field concerns sleep monitoring, where wearable devices and sensor networks enable continuous and non-intrusive assessment of sleep patterns and sleep quality in real-life conditions [29,30], also in the presence of pathologies.

The authors of Contribution 4 evaluate whether a commercially available smartwatch can accurately measure nighttime sleep outcomes in individuals with knee osteoarthritis and comorbid insomnia, comparing its performance to home-based polysomnography. The findings highlight the potential and limitations of wearable devices for continuous, non-intrusive sleep monitoring in real-life conditions; the device achieved high accuracy (85.76%) and sensitivity (95.95%), but low specificity (50.96%).

In Contribution 5, the authors investigate the impact of domain shift on predicting perceived sleep quality from wearable devices, showing how differences between populations or contexts can affect the accuracy of sleep quality predictions, and emphasizing the importance of personalized calibration for reliable sleep assessment, even if this presents some challenges (e.g., the need for user-provided labels). The authors also make a dataset available to the relevant research community.

Another important application domain treated in this Special Issue concerns the monitoring of older adults and individuals affected by neurodegenerative or mobility-impairing conditions. In these contexts, wearable sensors and distributed sensing systems enable the continuous assessment of mobility, fall risk, and functional status, supporting both clinical evaluation and independent living. Moreover, such technologies play a key role in Ambient Assisted Living (AAL) scenarios, where sensor-based monitoring can help detect early signs of frailty and support safer living conditions for people living independently at home [31,32].

The authors of Contribution 6 propose methods to enhance wearable fall detection systems using synthetic data, underlining the importance of reliable fall monitoring for older adults and individuals with mobility impairments in AAL environments. The idea is to use such data to supplement the limited availability of real data; in particular, the researchers extract fall segments from public footage and also generate synthetic fall signals via diffusion models. Personalization techniques would be needed to fit the models to individual behaviors.

In Contribution 7, the authors assess the agreement between optoelectronic systems and wearable sensors for the evaluation of gait spatiotemporal parameters in individuals with Progressive Supranuclear Palsy, highlighting how wearable devices can support continuous, objective mobility assessment in clinical and home settings. Indeed, the authors found a perfect agreement for gait speed and a good agreement for both cadence and gait cycle duration; they also provide suggestions to deal with eventual misalignments between test and reference systems.

Contribution 8 is a review providing an overview of assisted living solutions using wearable devices. The authors emphasize how distributed sensor networks can enable the early detection of frailty and support independent living for older adults. Particular attention is paid to electromyographic sensors and inertial measurement units (IMUs) for activity recognition along with physiological sensors to provide an overview of the psycho-physiological conditions of the subject. These technologies can greatly contribute to clinical best practices and to care management policies.

The authors of Contribution 9 explore physical frailty prediction through cane usage patterns (considering accelerations and angular velocities acquired through an IMU) during walking in older people with and without physical frailty. The performance of ML models is evaluated to distinguish between people with and without physical frailty, and an accuracy of 78.6% is achieved, demonstrating how subtle behavioral and mobility data captured by sensors can inform the personalized assessment of frailty risk.

Sensor-based systems can also be employed to investigate different aspects of human behavior, perception, and functional abilities. By exploiting wearable and embedded sensing technologies, it is possible to explore physiological responses, social interactions, and motor performance in real-world scenarios.

In Contribution 10, the authors investigated motion sickness by analyzing multimodal human signals collected in an autonomous driving simulator. They found a multimodal correlation between human signals and the perceived motion sickness level, showing how wearable sensors can capture physiological and behavioral responses in real-world scenarios to support personalized assessment of discomfort and adaptation.

A proximity sensor integrated with a Bluetooth sensor, a global positioning system (GPS), and an accelerometer are employed in Contribution 11 to measure social interactions in a school environment. The authors evaluated interaction through the analysis of neighboring sensors proximity, highlighting the potential of wearable and embedded sensors for monitoring human behavior and social dynamics in real-world settings.

The authors of Contribution 12 propose an automated architecture based on the Tapping Test protocol to assess hand dexterity through the usage of inertial sensors worn at finger level. Data processing provides useful information on the tests, demonstrating how wearable-based measurements can provide an objective, fine-grained evaluation of motor skills for clinical or research applications. Upper limb coordination and neuroplasticity aspects are also evaluated in an assessment of the function of motor abilities.

Finally, Contribution 13 focuses on sound direction detection and mixed source separation in embedded systems. Wearables today can have a speech recognition capability, which can be enhanced by an analysis of the auditory scene, going deeper into environmental perception in everyday contexts.

## 3. Conclusions

The contributions included in this Special Issue underline how wearable and distributed sensor systems enable continuous and non-intrusive monitoring while preserving user privacy, a key advantage over traditional approaches. By combining these sensors with AI-driven data analysis and human-in-the-loop paradigms, it is possible to extract meaningful insights across different domains, supporting more accurate, adaptive, and responsible interventions. These advancements highlight the potential of sensor technologies not only to improve wellbeing and safety but also to ensure ethical and privacy-conscious applications in real-world environments.

## Data Availability

Not applicable.
